# Supplementary material for: Dual targeting of Saccharomyces cerevisiae Pso2 to mitochondria and the nucleus, and its functional relevance in the repair of DNA interstrand crosslinks
Source: G3 (Bethesda). 2022 Apr 28;12(6):jkac066. doi: 10.1093/g3journal/jkac066 (PMC9157068; doi:10.1093/g3journal/jkac066)
Supplement: jkac066_Supplementary_Data [file jkac066_supplementary_data.pdf]

Supplementary material

Dual targeting of *Saccharomyces cerevisiae* Pso2 to mitochondria and the nucleus, and its functional relevance in the repair of DNA interstrand crosslinks

Shravanahalli C. Somashekara and Kalappa Muniyappa*
Department of Biochemistry, Indian Institute of Science, Bangalore 560 012

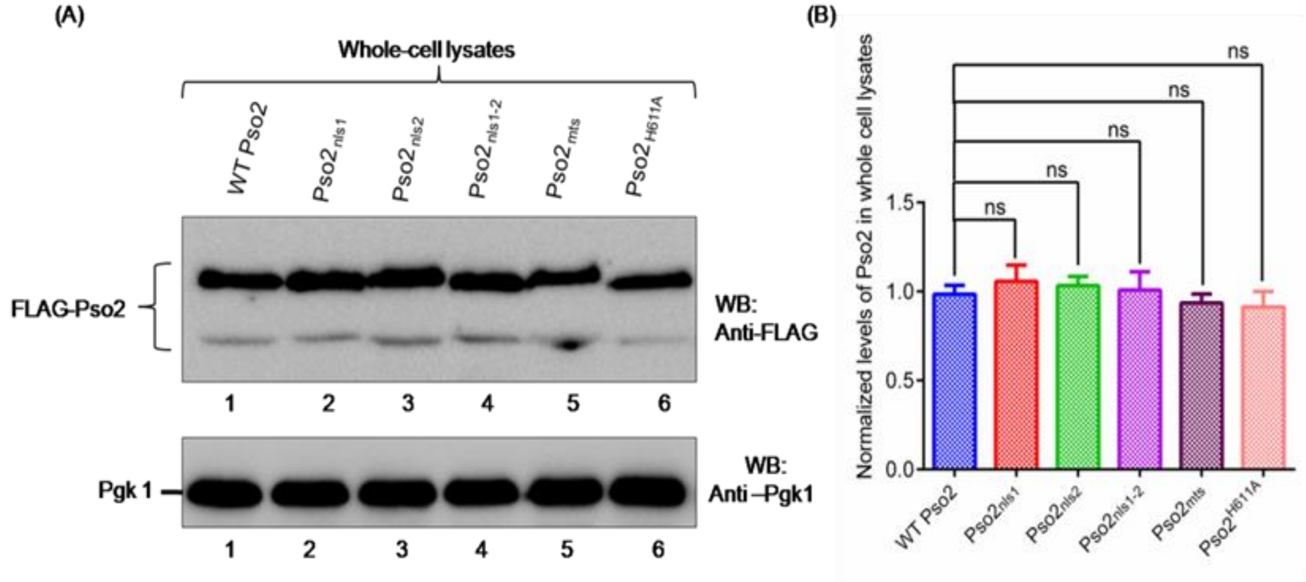

Figure S1. The levels of import-deficient Pso2 variants in the whole-cell lysates (A) Western blot analysis of whole-cell lysates of *S. cerevisiae pso2*Δ cells expressing Flag-tagged wild-type Pso2 and its import-deficient variants. (B) Quantification of relative levels of signal intensity shown in (A).The data is expressed as the mean ± SEM from three independent experiments. Statistical comparisons were performed by unpaired Student's t test. ns, not significant.
